# Integrated Analysis of Transcriptome and Metabolome and Evaluation of Antioxidant Activities in *Lavandula* *pubescens*

**DOI:** 10.3390/antiox10071027

**Published:** 2021-06-25

**Authors:** Chang Ha Park, Hyeon Ji Yeo, Ye Eun Park, Ye Jin Kim, Chanung Park, Jae Kwang Kim, Sang Un Park

**Affiliations:** 1 Department of Crop Science, Chungnam National University, 99 Daehak-ro, Yuseong-gu, Daejeon 34134, Korea; parkch804@gmail.com (C.H.P.); guswl7627@gmail.com (H.J.Y.); yeney1996@cnu.ac.kr (Y.E.P.); 2 Division of Life Sciences, College of Life Sciences and Bioengineering, Incheon National University, Yeonsugu, Incheon 22012, Korea; 201721047@inu.ac.kr; 3 Department of Botany and Plant Pathology, Purdue University, 915 West State Street, West Lafayette, IN 47907, USA; park947@purdue.edu; 4 Department of Smart Agriculture Systems, Chungnam National University, 99 Daehak-ro, Yuseong-gu, Daejeon 34134, Korea

**Keywords:** *Lavandula pubescens*, transcriptome, metabolome, antioxidant capacity

## Abstract

*Lavandula pubescens,* belonging to the Labiatae family, is a newly discovered strongly aromatic species of lavender that is potentially beneficial for human health. Given the economic importance of lavender species, we sought in this study to characterize the terpenoid biosynthesis of *L. pubescens* by obtaining transcriptomic and metabolic datasets. Transcriptome analysis of *L. pubescens* grown aseptically in tissue culture medium yielded 124,233 unigenes with an average length of 470 bp and N50 value of 522 bp from 9,476,122,928 raw reads. In order to provide relevant biological information, the unigenes were annotated using the following public databases: National Center for Biotechnology Information (NCBI) nucleotide (NT) and non-redundant protein (NR), Brassica (BRAD), Arabidopsis Information Resource (TAIR), Clusters of Orthologous Groups (COG), and Gene Ontology (GO). NR annotation results revealed that *L. pubescens* is genetically closely related to *Sesamum indicum.* On the basis of the transcriptome data, a total of 14 cDNA clones encoding the terpene biosynthetic genes *LpDXS*, *LpMCT*, *LpMCS*, *LpHDR*, *LpIDI*, *LpAACT*, *LpHMGS*, *LpHMGR*, *LpMVK*, *LpPMK*, *LpMVD*, *LpGPPS*, *LpSQS*, and *LpGGPPS* were identified in *L. pubescens*. These were quantified in the roots, stems, and leaves of *L. pubescens* using quantitative real-time polymerase chain reaction (qRT-PCR), which revealed that the gene expression levels were higher in the leaves and stems than in the roots, which was found to be consistent with the levels of ursolic and oleanolic acids in the different organs using high-performance liquid chromatography (HPLC). A total of 48 hydrophilic metabolites were identified and quantified in the organs using gas chromatography time-of-flight mass spectrometry (GC-TOFMS). Furthermore, the antioxidant activity of an ethyl acetate extract of *L. pubescens* leaves was examined using different methods to determine the potential therapeutic properties. A reducing power assay revealed that the absorbance values increased in a concentration-dependent manner, whereas a 2,2-diphenyl-1-picrylhydrazyl radical scavenging assay indicated the strong activity (60.4 ± 0.9%) of the ethyl acetate extract at a concentration of 100 µg/mL, which also showed strong hydrogen peroxide (57.4 ± 2.7%), superoxide radical (62.1 ± 0.7%), and hydroxyl radical (58.6 ± 0.4%) scavenging activities.

## 1. Introduction

**Figure 1:**
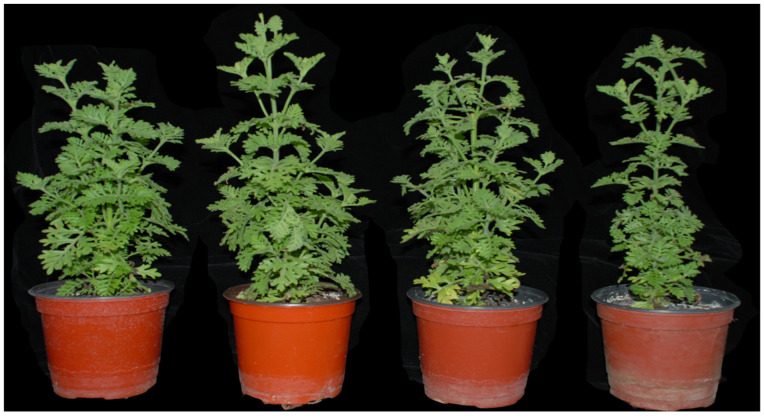
The phenotype of *L. pubescens* grown in a greenhouse.

Plants in the genus *Lavandula* (lavenders) within the family Lamiaceae (Labiatae), (which are native to the Arabian Peninsula, the Mediterranean, Russia, Southeast India, and Africa) are noted for their important medicinal properties [1]. There are currently 39 known species of lavender that can be classed based on the following sectional classification: section *Lavandula*; *Dentatae* Suarez–Cerv. and Seoane–Camba; section *Stoechas* Ging; section *Pterostoechas* Ging; section *Subnudae* Chaytor; section Chaetostachys Benth; and unclassified taxa [2]. Among the species in section *Pterostoechas*, *Lavandula pubescens* is a species native to the Yemen Arab Republic, Israel, Jordan, Saudi Arabia, Egypt, and Syria, and this aromatic plant is comprised of a distinctive stem with a long white stiff indumentum over a number of short-stalked glandular hairs, two to three pinnatisect leaves, and dense unbranched flower spikes [2,3]. The bracts are oval-shaped and slightly shorter or longer than the calyx. A wide variety of lavenders have similar chemical compositions and ethnobotanical properties [4]. The phenotype of *L. pubescens* grown in a greenhouse is shown in Figure 1. Moreover, they are characterized by the production of essential oils containing monoterpenes, diterpenes, sesquiterpenes, and aromachemicals, which are utilized in veterinary medicine, aromatherapy products, cosmetics, fragrances, and food processing [1,3]. Numerous lavender varieties have been developed and cultivated for a diverse range of medicinal, ornamental, and commercial purposes [1,3].

**Figure 2:**
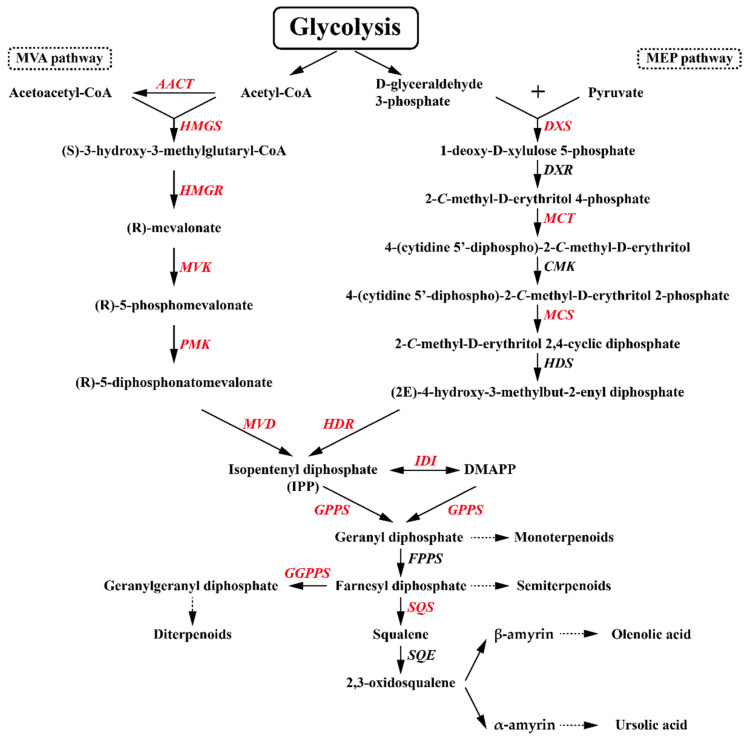
The proposed pathway for the biosynthesis of oleanolic acid and ursolic acid. The terpenoid biosynthetic genes identified in this study are indicated in red: MVA, mevalonic acid; MEP, 2-*C*-methyl-D-erythritol 4-phosphate; IPP, isopentenyl diphosphate; DMAPP, isomer dimethylallyl diphosphate; AACT, acetyl-CoA acetyltransferase; HMGS, hydroxymethylglutaryl-CoA synthase; HMGR, 3-hydroxy-3-methylglutaryl-CoA reductase; MVK, mevalonate kinase; PMK, phosphomevalonate kinase; MVD, mevalonate-5-pyrophosphate decarboxylase; DXS, 1-deoxy-D-xylulose-5-phosphate synthase; DXR, 1-deoxy-D-xylulose 5-phosphate reductoisomerase; MCT, 2-*C*-methyl-D-erythritol 4-phosphate cytidylyltransferase; CMK, 4-diphosphocytidyl-2-*C*-methyl-D-erythritol kinase; MCS, 2-*C*-methyl-D-erythritol 2,4-cyclodiphosphate synthase; HDS, 4-hydroxy-3-methylbut-2-enyl-diphosphate synthase; HDR, 4-hydroxy-3-methylbut-2-enyl diphosphate reductase; IDI, isopentenyl diphosphate isomerase; GPPS, geranyl diphosphate synthase; GGPPS, geranylgeranyl diphosphate synthase; FPPS, farnesyl pyrophosphate synthase; SQS, squalene synthase; and SQE, squalene epoxidase.

Terpenes are both the largest class of natural plant products and the most structurally diverse group in nature, the biosynthesis of which is initiated by the formation of two types of isoprene unit, isopentenyl diphosphate (IPP) and its isomer dimethylallyl diphosphate (DMAPP), derived from the mevalonate (MVA) or 1-deoxy-d-xylulose5-phosphate (MEP) pathway (Figure 2). In the subsequent metabolic step, geranyl diphosphate synthase (GPPS) catalyzes the formation of geranyl diphosphate (GPP) (a C_10_ precursor for monoterpene biosynthesis) via the condensation between IPP and DMAPP. The condensation between GPP and IPP is further catalyzed by farnesyl pyrophosphate synthetase (FPPS) to generate farnesyl pyrophosphate (FPP), known to be a C_15_ precursor of sesquiterpenes. The formation of geranyl geranyl diphosphate, also known as the C_20_ precursor of diterpenes, is catalyzed by geranyl geranyl diphosphate synthetase (GGPPS), which binds the resulting FPP with a further IPP. In the biosynthesis of triterpenes, there is a head-to-head dimerization between two FPP molecules, followed by an NADPH-requiring reductive rearrangement to form a squalene containing a C1-C1′ linkage between the two molecules, which is catalyzed by squalene synthase (SQS). Thereafter, squalene epoxidase (SQE) catalyzes the oxygenation of squalene to produce 2, 3-oxidosqualene, a common C_30_ precursor of triterpenes. Biosynthesis of oleanolic and ursolic acids involves the cyclization of 2,3-oxidosqualene to generate α-amyrin and β-amyrin, catalyzed by α-amyrin (aAS) and β-amyrin (bAS) synthases, respectively, which are successively 28-oxidized to ursolic acid and oleanolic acid via the activity of cytochrome P450 family members [5,6].

Advances in next-generation sequencing (NGS) technologies now enable cost-effective whole-transcriptome sequencing rather than whole-genome sequencing, with RNA-seq techniques providing functional information from genome transcripts [7]. Among the well-established NGS technologies, Illumina Solexa GA, one of the “Short-read” (36–72 bp) sequencing technologies, has been successfully applied in the whole-transcriptome analysis and de novo assembly of lavenders, including *L*. *angustifolia* ‘Lady’, *L*. *latifolia,* and their natural breed *L*. × *intermedia* ‘Grosso’ [8], and *L. angustifolia* ‘JX-2′ [9], and *L. angustifolia* ‘Maillette’ [10].

Free radicals, which are generated during normal cellular processes, are reactive oxygen species (ROS) that, in excess quantities, can potentially cause damage to nucleic acids, proteins, and lipids. To counter these detrimental effects, plants have evolved a range of antioxidant defense systems whereby antioxidants scavenge or deactivate these radicals [11]. However, under conditions where the balance between ROS production and deactivation is disrupted, ROS-induced oxidative stress can be sufficiently strong to cause irreparable damage [12]. From the perspective of human health, concerns regarding oxidative stress and the associated diseases and disorders have provided an incentive to increase the consumption of lavender plants or products, which may have beneficial effects with respect to the prevention of diverse diseases, including cancer and diabetes [3,13,14,15]. Moreover, lavender plants are also noted for their antimicrobial properties that can contribute to inhibiting pathogen growth [16]. These beneficial effects, associated with antioxidative, antidiabetic, and anti-inflammatory properties, are attributed to a diverse range of metabolites, including terpenes, phenolic compounds, flavonoids, anthocyanins, and carotenoids [4,17,18,19].

To date, there have been few studies that have focused on the RNA-seq analysis and metabolic profiling of *L*. *pubescens*. In this study, we employed the Illumina NGS platform to provide biological information on *L. pubescens* and examine the terpenoid biosynthetic pathway in this species. We also used qRT-PCR-based transcriptomic profiling and HPLC- and GC-TOFMS-based metabolite profiling to characterize triterpenoid biosynthesis in different parts of the *L. pubescens* plant and evaluated the antioxidant potential of *L. pubescens* leaf. The aim of this study was to compile important datasets acquired from the comprehensive analysis of the transcriptome and metabolome of *L. pubescens* (thereby providing a basis for future studies) and to characterize terpenoid biosynthesis in the different organs of *L. pubescens*.

## 2. Materials and Methods

### 2.1. Plant Materials and Sample Preparation for RNA Sequencing

Seeds of *L. pubescens* were aseptically germinated and grown in a half-strength Murashige and Skoog medium under a 16-h photoperiod at 25 °C in a growth chamber equipped with standard cool white fluorescent tubes with a flux rate of 35 μmol·s^−1^·m^−2^. After 2 months, whole plants were harvested, frozen in liquid nitrogen, and powdered for RNA extraction. RNA was isolated using a cetyltrimethylammonium bromide (CTAB)-based RNA isolation protocol in conjunction with a Plant RNA Mini Kit (Geneaid, Sijhih, Taiwan). The quality (A260/A280 ratio) and quantity of the total RNA thus prepared were measured using a NanoVue Plus spectrophotometer (GE Healthcare, Chalfont Saint Giles, UK), and RNA integrity was assessed using a 1.0% denaturing agarose gel.

### 2.2. Illumina RNA Sequencing

The whole procedure was performed with our previously described method [20]. Sequencing of *L. pubescens* RNA was conducted using Set A and Set B of a TruSeq Stranded Total RNA LT Sample Prep Kit with Ribo-Zero Gold (Set A; Illumina, RS-122-2301 and Set B; Illumina, RS-122-2302, San Diego, CA, USA). Initially, ribosomal RNA (rRNA) was removed from total RNA using biotinylated and target-specific oligos and rRNA removal beads, and the purified RNA thus obtained was sheared into small fragments (100–400 bp) using divalent cations at 94 °C for 8 min. Oligo (dT)-dynabead selection was then performed to isolate poly (A) mRNA. First-strand cDNA was synthesized from the mRNA using random hexamer primers and reverse transcriptase, with the cleaved RNA fragments as templates. Prior to second-strand synthesis, the RNA templates were cleaned using RNase H and the second strand was synthesized by incorporating dUTP instead of dTTP to yield a double-stranded cDNA, which was subsequently 3′-adenylated and subjected to adapter ligation. The final step involved size selection of the resulting products, based on gel electrophoresis and polymerase chain reaction (PCR) amplification to generate the final cDNA library. The library was sequenced via paired-end (PE) flow cell Illumina using a NextSeq 500 sequencer (Illumina, San Diego, CA, USA) to generate raw reads. The raw *L. pubescens* RNA sequencing reads were preprocessed using both FastQC and CutAdapt adapter software [21] to generate high-quality cleaned reads by eliminating low-quality reads containing unknown base N, reads with more than 10% *Q* < 20 bases, and adapter sequences, given that low-quality and adapter sequences in the raw reads might cause errors during further de novo assembly. As a consequence of adapter and quality trimming, a total of 124,685,828 high-quality clean reads were prepared for the *L. pubescens* transcriptome.

### 2.3. De Novo Assembly of L. pubescens Unigenes

The Trinity de novo assembly program with fixed default parameters was used to reconstruct full-length transcripts by combining the overlapping trimmed reads into contigs without gaps [22]. The properties of the assembled contigs, including average, maximum, minimum, median, and N50 lengths, were then calculated using the Trinity software package [23], after which the contig sequences were clustered based on sequence similarity and individual clusters were represented by an isoform set for the sequence of an expressed gene using CD-HIT-EST software [24]. The longest sequences were selected as unigenes for the corresponding genes of *L. pubescens*.

### 2.4. Annotation of the L. pubescens Unigenes

Using the unigene sequences obtained, we searched a number of public nucleotide and protein databases to identify the *L. pubescens* unigenes and elucidate their biological functions. BLASTN searches were conducted using the NCBI nucleotide nonredundant nucleotide sequence (NT) database using a cutoff of e-value < 1.0 × 10^−5^. A further five public databases, namely, the NCBI NR, SWISS-PROT (UniProt) protein sequence, BRAD, TAIR, and COG databases, were screened using the same cutoff value (e-value < 1.0 × 10^−5^) to identify protein coding sequences in genomic DNA or proteins encoded by transcripts. On the basis of SWISS-PROT annotation, the *L. pubescens* unigenes were assigned to the GO terms molecular function, biological processes, and cellular components using the Blast2GO program. Having obtained GO annotations for all unigenes, we used WEGO software to perform GO functional classification analyses for all unigenes and to establish the distribution of gene functions at the macro level [25]. The assembled transcripts were deposited in the NCBI Short Read Archive database (SRA, http://www.ncbi.nlm.nih.gov/Traces/sra/, accessed on 22 May 2021). The accession number was SRR14621901.

### 2.5. Identification of Genes Associated with Terpene Biosynthetic Pathways

On the basis of the transcriptome data obtained, we identified candidate genes involved in terpene biosynthesis, which we confirmed by performing either nucleotide or protein homology searches of the NCBI GenBank database (http://www.ncbi.nlm.nih.gov/BLAST, accessed on 25 June 2021).

### 2.6. Plant Materials for Triterpenoid HPLC and Gene Expression Analyses

*L. pubescens* plants used for triterpenoid HPLC and gene expression analyses were harvested from the experimental plantation of Chungnam National University, with four biological replicates being used for each analysis. Different plant parts (roots, leaves, and stems) were harvested in August 2019 and immediately immersed in liquid nitrogen, subsequently being ground finely with liquid nitrogen. The powders thus obtained were used for quantitative real-time polymerase chain reaction (qRT-PCR) analysis of terpenoid biosynthetic genes, whereas the remaining samples were freeze-dried for the triterpenoid HPLC analysis.

### 2.7. RNA Extraction and cDNA Synthesis

Total RNA was extracted from individual organs using a CTAB-based RNA isolation protocol in conjunction with a Plant RNA Mini Kit (Geneaid, Sijhih City, Taiwan) and the addition of DNase, according to the manufacturer’s instructions. The quality and quantity of the isolated RNA were determined using a NanoVue Plus spectrophotometer, and RNA integrity was assessed by running samples on a 1.0% denaturing agarose gel. Aliquots (1 μg) of the total RNA obtained from different plant parts was subjected to reverse transcription using a Superscript II First Strand Synthesis Kit (Invitrogen; Carlsbad, CA, USA) and oligo (dT)_20_ primers according to the manufacturer’s protocol.

### 2.8. Quantitative Real-Time PCR

The method and conditions used for real-time PCR have been described in a previous study [26]. For the qRT-PCR assay, sets of gene-specific primers for terpenoid biosynthesis were designed using the Gene Runner program (version 3.05). A 20-fold dilution of the cDNA product was subjected to PCR amplification in triplicate in a CFX96 Real-Time System combined with a C1000 Thermal Cycler (Bio-Rad, Hercules, CA, USA) using a Quantitect SYBR Green PCR Kit (Qiagen, Hilden, Germany). Assays were carried out using 20-μL qRT-PCR reaction mixtures comprised of 5 μL of cDNA, 0.5 μM of each specific primer, and 10 μL of 2× SYBR Green Real-time PCR Master Mix, and made up to the final volume using DEPC-treated water. The PCR reactions were performed using the following thermal cycling conditions: predenaturation at 95 °C for 15 min, followed by 40 cycles of denaturation at 95 °C for 15 s, annealing at 55 °C for 15 s, and extension at 72 °C for 20 s. Each assay was run with a negative control, with water being used instead of cDNA, and a series of standards. Three replicates for each hairy root line sample were analyzed using Bio-Rad CFX Manager 2.0 software (Bio-Rad, Hercules, CA, USA). The expression of terpene biosynthesis genes was calculated using the 2^−∆Ct^ method [27].

### 2.9. Triterpenoid HPLC Analysis

Triterpenoids (oleanolic acid and ursolic acid) were extracted using a previously reported method with slight modifications [28]. Fine powders (100 mg) of different parts (roots, stems, and leaves) of *L. pubescens* plants were suspended in 2 mL of methanol (95% *v*/*v*) containing 1% HCl and then sonicated for 60 min. The sonicated samples were then centrifuged and the resulting supernatants collected. The remaining sludge was re-extracted twice in the same manner. The combined supernatants were dried using nitrogen gas and then dissolved in methanol (0.5 mL). HPLC analysis was performed using an Agilent 1200 series HPLC system incorporating a Welch Ultisil PAH column (4.6 × 250 mm, 5 μm, Welch Materials, West Haven, CT, USA) to separate the oleanolic and ursolic acids. The mobile phase consisted of acetonitrile and water (85:15) at a flow rate of 1 mL/min. The oven temperature was set to 30 °C, and the detection wavelength was 210 nm. Calibration curves were constructed by plotting six different concentrations of oleanolic and ursolic acids. The linear equations for oleanolic acid and ursolic acid were y = 1.1342 x + 0.5792 (R^2^ = 0.9999) and y = 1.7683 x + 0.0763 (R^2^ = 0.9998), respectively.

### 2.10. Total Phenolic and Flavonoid Contents

The total phenolic and flavonoid contents of the methanol extracts prepared from each sample were determined using methods described in our previous study [29]. Fine powders (100 mg) prepared from the different plant parts were extracted with 3 mL of methanol and sonicated for 1 h. The extract was then centrifuged at 20,929× *g*, filtered into a vial through a 0.45-µm PTFE hydrophilic syringe filter, and used for further experiments to evaluate the total phenolic and flavonoid contents. For the assay of total phenolic contents, 0.1 mL of sample extract was mixed with 3 mL of ultrapure water, followed by the addition of 0.5 mL of 2N Folin and Ciocalteu phenol reagent (Sigma-Aldrich Co., Yongin, Korea). Having incubated the mixture for 3 min at room temperature, 2 mL of sodium carbonate (20%, *w*/*v*) was added, and following a further 60-min incubation in the dark, the absorbance of each sample was determined at 760 nm. An equivalent calibration curve (standard curve equation: y = 0.0013 x + 0.0313, R^2^ = 0.9989) was prepared using different concentrations of gallic acid solution ranging from 12.5 to 250 μg/mL. The results are presented in terms of milligrams of gallic acid equivalent per gram of dry weight (mg gallic acid equivalent (GAE)/g dry weight (DW)). For the total flavonoid content assay, 0.1 mL of sample extract was mixed with 4 mL of ultrapure water, followed by the addition of 0.3 mL of NaNO_2_ (5%, *w*/*v*). After incubating for 5 min at room temperature, 0.3 mL of AlCl_3_ (10%, *w/v*) was added, followed by incubation for 6 min and the subsequent addition of 2 mL of 1 M NaOH. The final volume of the mixture was brought to 10 mL with ultrapure water. An equivalent calibration curve (standard curve equation: y = 0.0001 x + 0.0380, R^2^ = 0.9997) was prepared using different concentrations of rutin solution ranging from 62.5 to 1000 μg/mL. The results are expressed in terms of milligrams of rutin equivalent per gram of dry weight (mg Rutin equivalent (RE)/g dry DW).

### 2.11. GC-TOFMS Analysis

The hydrophilic metabolites were extracted and analyzed according to previously described methods [30]. Fine powders (10 mg) of the different parts (roots, stems, and leaves) of *L. pubescens* were mixed with 1 mL of water-chloroform-methanol mixture (1:1:2.5 *v*/*v*/*v*). The addition of 60 µL of ribitol (0.2 g/L), as an internal standard, was performed. After mixing at 1200× *g* and 37 °C for 30 min in a compact thermomixer and then centrifuging at 11,000× *g* for 10 min, the polar phase (0.8 mL) was moved into a fresh tube containing 0.4 mL of laboratory grade water and then the mixtures were evaporated in a SpeedVac vacuum concentrators for 3h. Subsequently, the derivatization with 80 μL of methoxyamine hydrochloride/pyridine (20 g/L) was carried out and then shaken at 37 °C for 2 h. Afterwards, 80 μL of N-methyl-N-(trimethylsilyl)trifluoroacetamide was added, followed by heating at 37 °C for 30 min. After spinning down, the final extract was transferred to a GC vial. The analysis equipment, condition, and gradient program of GC-TOFMS were used according to our previous study [30]. Quantification was performed using selected ions, and the Chroma-TOF software was used to locate peaks.

### 2.12. Antioxidant Assays

#### 2.12.1. Reducing Power Activity

The reducing power of the *L. pubescens* extract was assessed using a method reported in our previous study [31]. Assay mixtures were prepared using 2.5 mL of 0.2 M phosphate buffer (pH 6.6) and different concentrations of extract (20–100 µL) in 15-mL falcon tubes, followed by the addition of 2.5 mL of 1% K_3_Fe(CN)_6_. The mixtures were incubated at 50 °C for 20 min and placed in tubes, and following the addition of 2.5 mL of 10% TCA, were centrifuged at 3000 rpm for 10 min. A 2.5-mL volume of the resulting supernatant was mixed with 2.5 mL of distilled water and 0.5 mL of 1% FeCl_3_, and thereafter, the absorbance was determined at 700 nm. An increase in the absorbance of the reaction mixture was taken to be indicative of an increase in reducing power. As a standard, we used ascorbic acid, and a sample containing water instead of extract was used as a blank. The experiment was performed in triplicate and average values were used for analysis. A standard ascorbic acid stock solution of 1 mg/mL was prepared for all experiments.

#### 2.12.2. 2,2-Diphenyl-1-picrylhydrazyl (DPPH) Radical Scavenging Assay

The DPPH scavenging activity of *L. pubescens* extracts was evaluated using a method reported in a previous study [31]. We initially prepared a 0.15% DPPH solution in cold methanol. The reaction mixtures consisted of 3.8 mL of methanol and different concentrations of extracts (20–100 µL) to which 200 µL of DPPH solution was added, followed by incubation in the dark at room temperature for 30 min. Absorbances were subsequently determined at 517 nm, with ascorbic acid used as a standard.

#### 2.12.3. Hydrogen Peroxide Scavenging Activity

The hydrogen peroxide (H_2_O_2_) scavenging activity of an ethyl acetate extract of *L. pubescens* was evaluated using a previously reported method [31]. A 40 mM solution of H_2_O_2_ was prepared in phosphate buffer (pH 7.4). The reaction mixtures contained different concentration of extract (20–100 µL) and 1 mL of distilled water, to which 0.6 mL of the H_2_O_2_ solution was added, followed by incubation for 10 min at room temperature and subsequent determination of the absorbance at 560 nm, with ascorbic acid used as a standard.

#### 2.12.4. Superoxide Radical Scavenging Activity

The superoxide scavenging activity of extracts was measured using nitroblue tetrazolium (NBT) according to a previously reported method [31]. Nitrate reduction occurs during the generation of superoxide radicals via the oxidation of hydroxyl amine hydrochloride in the presence of NBT. To the *L. pubescens* extracts (20–100 µL), we added 1 mL water, 100 µL of 24 mM NBT, and 0.2 mL of 0.1 mM EDTA. Reactions were initiated by the addition of 0.1 mL of 1 mM hydroxylamine hydrochloride, followed by incubation at 25 °C for 15 min. The reduction of NBT was measured at 560 nm, with a no-extract sample being used as a control.

#### 2.12.5. Hydroxyl Radical Scavenging Activity

The hydroxyl scavenging assay of *L. pubescens* extracts was performed as described previously [31]. The reaction mixtures consisted of 100 µL of 28 mM 2-deoxy-2-ribose (dissolved in phosphate buffer, pH 7.4), 20–100 µL of *L. pubescens* extracts (made up to 100 µL with distilled water), 200 µL of 100 mM FeCl_3_ and 100 mM EDTA (1:1 *v*/*v*), 100 µL H_2_O_2_ (1 mM), and 100 µL ascorbic acid (1 mM). Following a 1-h incubation at 37 °C, the extent of deoxyribose degradation was assessed based on the TBA reaction, with absorbance being read at 532 nm against a blank solution. Ascorbic acid was used as a positive control.

### 2.13. Statistical Analysis

Duncan’s multiple range test (*p* < 0.05) was performed using Statistical Analysis System software (SAS, system 9.4, 2013; SAS Institute, Inc., Cary, NC, USA), whereas principal component analysis (PCA), partial least-squares discriminant analysis (PLS-DA), hierarchical clustering analysis (HCA) based on Pearson correlation results, and heatmap visualization was carried out using MetaboAnalyst 5.0 (http://www.metaboanalyst.ca/, accessed on 3 May 2021) with auto-scaling (unit variance scaling).

## 3. Results

### 3.1. Transcriptome Sequencing and De Novo Assembly of L. pubescens

After trimming adaptor and low-quality sequences with more than 10% Q < 20 bases or ambiguous N bases, we obtained a total of 124,685,828 clean reads, with more than 89.31% of the bases having a quality score of Q > 30 and mean quality score of 34.41, comprising 9,476,122,928 nucleotides (9.47 Gb). The clean reads were assembled de novo into 180,249 contigs with average, maximum, median, and minimum lengths of 480, 11,308, 322, and 224 bp, respectively, and an N50 value of 545 bp (Table S1). Using the Trinity program, we assembled a total of 124,233 unigenes with average, maximum, median, and minimum sizes of 470, 11,308, 319, and 224 bp, respectively, and an N50 value of 522 bp (Table S1). In terms of *L. pubescens* unigene size distribution, 74.83% (92,967) of the 124,233 unigenes were less than 500 nt in length, 17.21% (21,387) were between 500 and 1000 nt, 7.67% (9533) were between 1000 and 3000 nt, and 0.28% (346) were more than 3000 nt (Figure S1).

### 3.2. Functional Annotation and Classification of L. pubescens Unigenes

**Figure 3:**
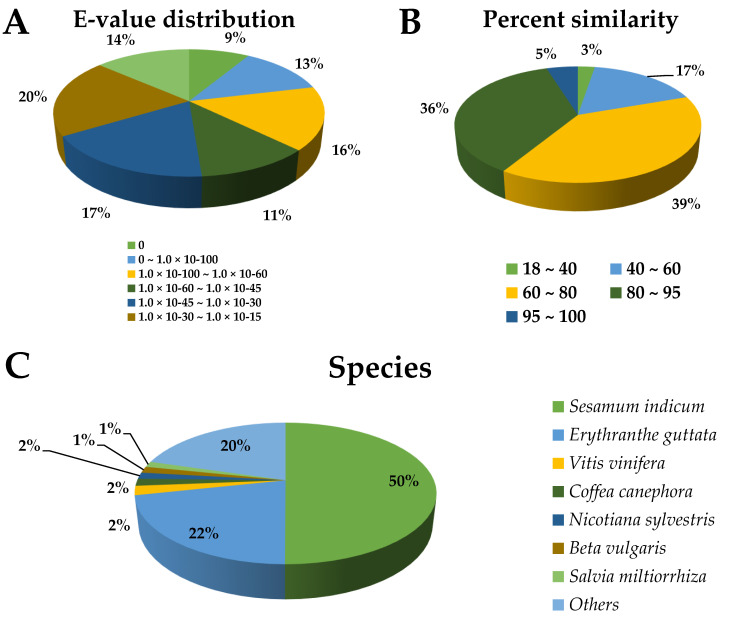
e-value distribution (**A**), percentage similarity (**B**), and species distribution (**C**) of the nonredundant (NR) annotation results for *Lavandula pubescens* unigenes.

To obtain information regarding the function of *L. pubescens* unigenes, we performed annotations based on sequence alignments with those in multiple public databases (NR, NT, SWISS-PROT, BRAD, TAIR, COG, and GO) with an e-value cutoff of 1 × 10^−5^. A total of 68,695 unigenes (55.30% of the 124,233 unigenes) were successfully matched with sequences in the databases: 64,993 (52.32%), 20,947 (16.86%), 45,750 (36.83%), 57,951 (46.65%), 56,604 (45.56 %), 16,019 (12.89%), and 44,814 (36.07%) unigenes in the NR, NT, SWISS-PROT, BRAD, TAIR, COG, and GO databases, respectively (Table S2). The e-value distribution in NR annotation indicated that 37.3% of the *L. pubescens* unigenes showed strong homology (e-value < 1 × 10^−60^) (Figure 3A) with database sequences, and percentage similarity distributions indicated that 41% of the given unigenes had a high similarity (>80%), whereas the remaining 59% of the matched sequences showed a similarity between 18% and 60% (Figure 3B). In terms of species distribution (Figure 3C), 50.05% of the *L. pubescens* unigenes had matches with genes in *Sesamum indicum*, followed by *Erythranthe guttata* (21.56%), *Vitis vinifera* (2.12%), *Coffea canephora* (1.64%), *Nicotiana sylvestris* (1.46%), *Beta vulgaris* (1.45%), and *Salvia miltiorrhiza* (1.30%).

COG annotation is considered a standard procedure for determining the function and evolution of coding proteins at the genome scale. On the basis of COG annotation, a total of 16,019 unigenes were functionally predicted and classified into 26 categories, the largest of which was “Translation, ribosomal structure and biogenesis” (1799 COG-annotated unigenes, 11.36%), followed by “Carbohydrate transport and metabolism” (1590, 10.04%), “Post-translational modification, protein turnover, chaperones” (1464, 9.24%), “General functional prediction only” (1441, 9.10%), and “Signal transduction mechanisms” (1330, 8.40%) (Figure S2).

GO standard systems have been introduced to comprehensively delineate the functions of the predicted unigenes and their products. In the present study, we grouped *L. pubescens* unigenes into the three major GO categories; namely, cellular component, molecular function, and biological process, with 51 function sections, as shown in Figure S3. With respect to the cellular component category, the unigenes matched 14 terms, among which most unigenes were found to be associated with cells and cell parts. Similarly, unigenes were matched to 14 terms in the molecular function category, the majority of which were found to be associated with binding and catalytic processes. In biological process category, unigenes were assigned to 23 terms, with most being associated with cellular and metabolic processes (Figure S3).

### 3.3. Expression Analysis of Terpene-Related Genes in Different Organs of L. pubescens

**Figure 4:**
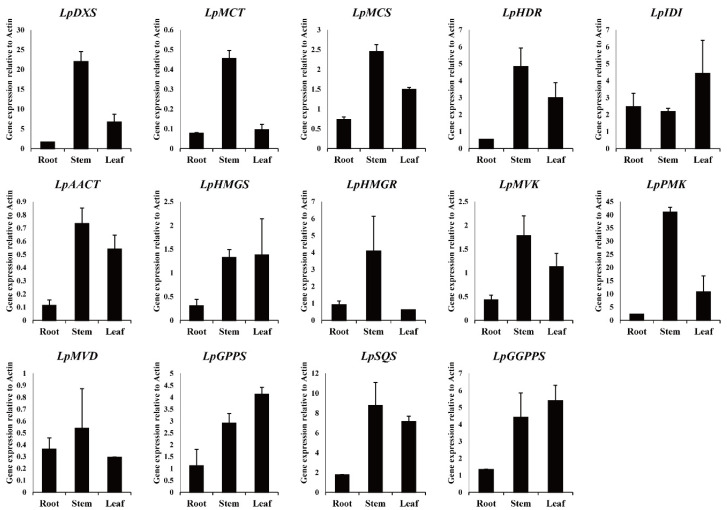
Expression of terpene biosynthetic genes in different organs of *Lavandula pubescens*.

Candidate terpene biosynthetic genes identified in the *L. pubescens* transcriptome were confirmed through NCBI homology BLAST searches, showing a high identity with other orthologous genes and proteins from a broad range of plant species. These genes were designated *LpDXS*, *LpMCT*, *LpMCS*, *LpHDR*, *LpIDI*, *LpAACT*, *LpHMGS*, *LpHMGR*, *LpMVK*, *LpPMK*, *LpMVD*, *LpGPPS*, *LpSQS*, and *LpGGPPS (*Table S3). On the basis of the information presented in Table S3, we designed specific primers and used these for real-time PCR (Table S4) to characterize terpene synthesis in *L. pubescens,* with *LpActin* being used as a reference gene for normalization. The analysis revealed that the expression levels of *LpDXS*, *LpMCT*, *LpMCS*, *LpHMGR*, and *LpPMK* were higher in stems than in roots and leaves, whereas those of *LpHDR*, *LpAACT*, *LpHMGS*, *LpMVK*, *LpSQS*, and *LpGGPPS* were higher in the stems and leaves, and *LpGPPS* and *LpIDI* levels were higher in the leaves (Figure 4).

### 3.4. Oleanolic Acid and Ursolic Acid Contents in Different Organs of L. pubescens

HPLC analysis confirmed the presence of oleanolic and ursolic acids in the roots, stems, and leaves of *L. pubescens* (Table 1), with the highest levels of oleanolic acid being detected in the stem and the highest levels of ursolic acid detected in the stem. Specifically, the levels of oleanolic acid and ursolic acid in root were 11.2- and 1.25-fold and 14.6- and 1.08-fold higher than those in the leaves and stems, respectively.

### 3.5. Total Phenolic and Total Flavonoid Contents

As indicated in Table 2, leaves were found to have the highest total phenolic content among the plant parts assessed (9.58 ± 0.19 mg GAE/g DW), followed by roots (8.08 ± 0.11 mg) and stems (3.83 ± 0.07 mg). Similarly, the leaves contained the highest amounts of flavonoids (29.56 ± 0.51 mg RE/g dry DW), which was 2.52-fold higher than the lowest level in stems (11.71 ± 0.18 mg).

### 3.6. GC-TOFMS Analysis

Identification and quantification of metabolites from the roots, stems, and leaves of *L*. *pubescens* were carried out. A total of 48 metabolites (one amine, two phenolic acids, two carbohydrate intermediates, four sugar alcohols, four citric acid cycle (CAC) intermediates, seven carbohydrates, 18 amino acids, and 10 organic acids) were detected in the different plant parts of *L*. *pubescens* (Figure S4). Among the CAC cycle intermediates, the levels of citric acid, malic acid, and fumaric acid were higher in the leaves and roots of *L*. *pubescens*. Similarly, organic acid levels were higher in the leaves and roots. Shikimic acid, glyceric acid, and threonic acid levels were higher in the leaves and lactic acid, oxalic acid, and glycolic acid levels were higher in the roots. Pyruvic acid levels were higher in the leaves and roots. Among the carbohydrates and their derivatives, the levels of fructose, mannose, galactose, glucose, and fructose-6-phosphate, excluding sucrose, were higher in the stems and roots. In contrast, the levels of sugar alcohols were higher in the leaves. For the amino acid biosynthesis, most amino acid levels were higher in leaves and stems. The levels of aspartic acid, glutamic acid, phenylalanine, and tyrosine were higher in leaves and the levels of pyroglutamic acid, 4-aminobutyric acid, asparagine, and glutamine were higher in the stems.

**Figure 5:**
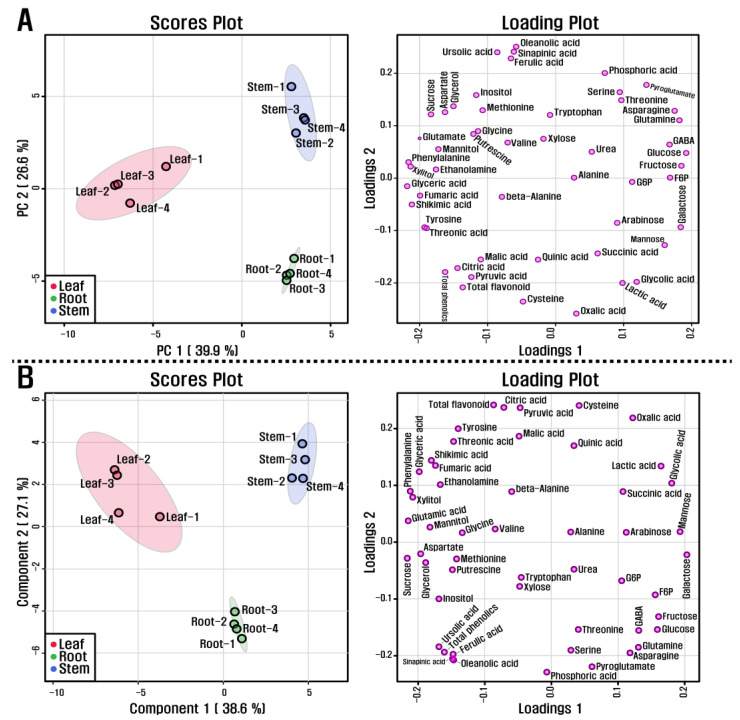
(**A**) scores and loading plots of PCA model and (**B**) scores and loading plots of PLDA model obtained from metabolites detected in different organs of *L*. *pubescens* using HPLC and GC-TOFMS.

PCA supported the above description on metabolite differences in the roots, stems, and leaves of *L*. *pubescens*. The PCA created a visualization of this metabolite data of the different plant parts of *L*. *pubescens* with two components (39.9 and 26.6% of the variance, respectively) in Figure 5A. Specifically, principal component 1 (PC1) displayed that the leaf group was separated from the stem and root groups, but the stem group was not separated from the root group and PC2 indicated a clear separation between the root and stem group. The most important metabolites of PC1 were glyceric acid, phenylalanine, xylitol, shikimic acid, and glutamic acid for which the eigenvector values were −0.21639, −0.21438, −0.21136, −0.20955, and −0.19997, respectively, and glucose, fructose, galactose, glutamine, and asparagine, for which the values were 0.19391, 0.18685, 0.1859, 0.18396, and 0.17692, respectively. These metabolites resolved this separation among the groups. In addition, PLS-DA, used to maximize the separation between groups, exhibited a clear separation between the roots and stems of *L*. *pubescens* in its two components, explaining 38.6 and 27.1% of the variance, respectively (Figure 5B).

**Figure 6:**
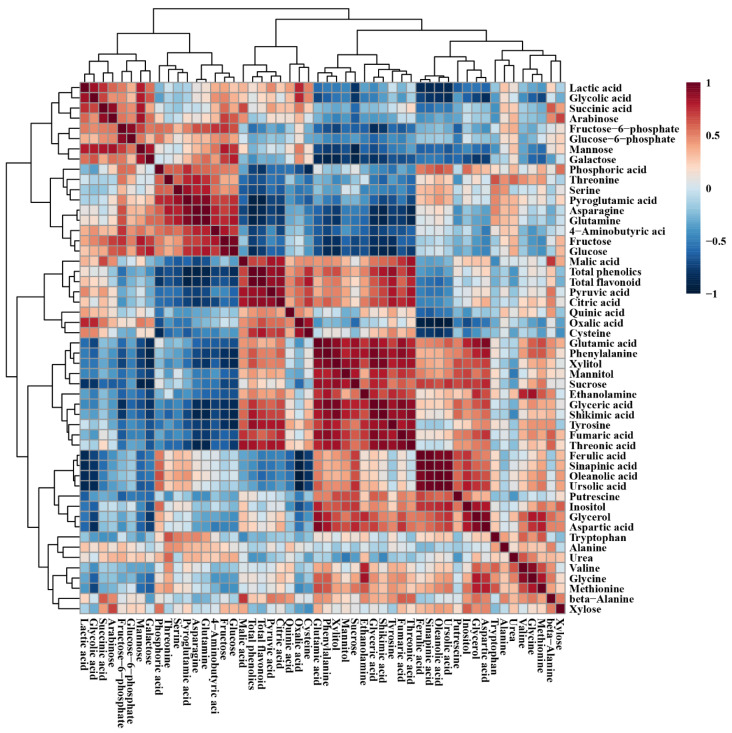
Correlation matrix of metabolites detected in different organs of *L*. *pubescens*. Each square indicates the Pearson’s correlation coefficient of a pair of compounds, and the value of the correlation coefficient is represented by the intensity of blue or red colors, as indicated on the color scale.

The relationship between the metabolites detected in the roots, stems, and leaves of *L*. *pubescens* was investigated using Pearson’s correlation (Figure 6). Phenylalanine, biosynthesized from shikimic acid, was positively correlated with shikimic acid (*r* = 0.890, *p* = 0.000) and phenylalanine, an initial precursor for the phenolic compounds, was positively correlated with total phenolic content (*r* = 0.630, *p* = 0.028). Furthermore, glucose-6-phosphate was positively correlated with fructose-6-phosphate (*r* = 0.891, *p* = 0.000). Among the CAC cycle intermediates, citric acid showed a strong correlation with malic acid (*r* = 0.806, *p* = 0.001) and fumaric acid (*r* = 0.751, *p* = 0.005). Sucrose was positively correlated with the detected secondary metabolites, including ferulic acid (*r* = 0.696, *p* = 0.012), sinapinic acid (*r* = 0.673 *p* = 0.016), oleanolic acid (*r* = 0.638 *p* = 0.026), and ursolic acid (*r* = 0.738 *p* = 0.006).

### 3.7. In-Vitro Assays of L. pubescens Antioxidant Activity

In this study, among the organs assessed, we established that the leaves contain the high amounts of triterpenoids, total phenolic content, and total flavonoid content, and on the basis of these observation, we sought to determine the antioxidant ability of *L. pubescens* leaf extracts.

**Figure 7:**
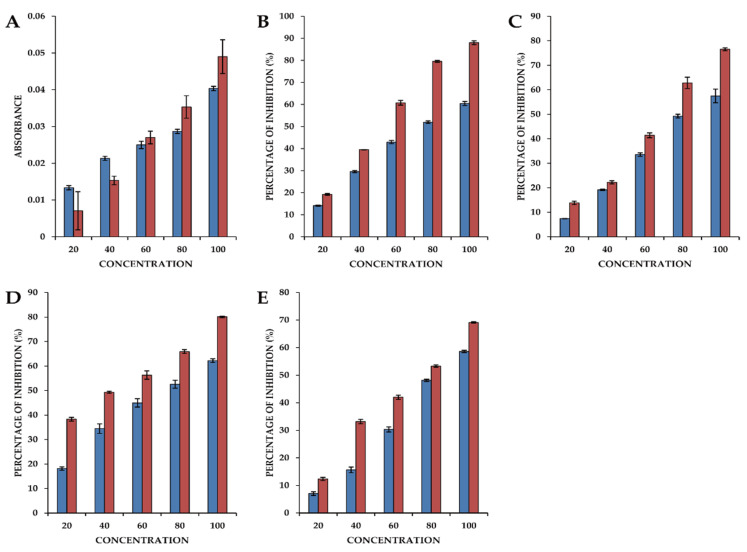
The antioxidant activity of an ethyl acetate extract of *Lavandula pubescens* leaves. (**A**) Reducing power activity; (**B**) 2,2-diphenyl-1-picrylhydrazyl (DPPH) radical scavenging activity; (**C**) Hydrogen peroxide scavenging activity; (**D**) Superoxide radical scavenging activity; (**E**) Hydroxyl radical scavenging activity. The blue bars represent values obtained using an ethyl acetate extract of *L. pubescens* leaves and the red bars are values obtained using ascorbic acid.

The reducing power of a chemical compound is considered an important indicator for antioxidant screening, as it is indicative of electron transfer capacity. These reducers can promote the conversion of the Fe^3+^/ferricyanide complex to the Fe^2+^/potassium ferrocyanide form, which thereby provides a basis for assaying antioxidant activity. In the present study, we assessed the reducing power activity of a crude *L. pubescens* ethyl acetate extract, which was compared with that of ascorbic acid (Figure 7A). We accordingly established that the extract has significant reducing power, as indicated by a concentration-dependent increase in the absorbance of the reaction mixture. Indeed, even at low concentrations, the antioxidant capacity of the extract was higher than that of ascorbic acid.

An assessment of DPPH radical scavenging activity is one of the most efficient methods for screening the antioxidant activity of plant extracts, and we accordingly observed that the antioxidant activity of the ethyl acetate extract of *L. pubescens* was comparable to that of ascorbic acid used as a standard and was concentration dependent, with a highest scavenging activity of 60.46 ± 0.9% being obtained using the extract at a concentration of 100 µg/mL (Figure 7B).

Hydrogen peroxide can rapidly cross cell membranes, and having entered the cytoplasm may react with Fe^2+^ ions and possibly Cu^2+^ ions to form hydroxyl radicals, which are potential origins of many of its toxic effects. We found that the H_2_O_2_ radical scavenging activity of the *L. pubescens* ethyl acetate extract was concentration dependent and comparable to that of the ascorbic acid standard, with the highest activity of 57.46% being obtained using the extract at 100 µg/mL, which compares with the activity of 76.52% obtained with ascorbic acid used at a concentration of 80 µg/mL (Figure 7C).

Superoxide radical scavenging activity was assessed by calculating the percentage inhibition of superoxide radical generation by the *L. pubescens* ethyl acetate extract compared with that obtained using the standard ascorbic acid. At an extract concentration of 80 µg/mL, we obtained a superoxide radical scavenging activity of approximately 50%, which increased to 62.6% when using the extract at a concentration at 100 µg/mL. Comparatively, the highest scavenging activity using ascorbic acid was 80.07% obtained using a concentration of 100 µg/mL (Figure 7D).

The hydroxyl radical (•OH) is particularly reactive in biological systems and has been associated with highly damaging species in free radical pathology and damaging biomolecules in living cells. These radicals combine with nucleotides in DNA, thereby promoting strand breakage and consequent carcinogenic, mutagenic, and cytotoxic effects. The hydroxyl radical scavenging capacity of an extract directly reflects its antioxidant activity, and in the present study, the highest level of antioxidant activity (58.62%) was obtained using the 100 µg/mL *L. pubescens* extract, which compares with highest value of 69.10% obtained using 100 µg/mL ascorbic acid (Figure 7E).

## 4. Discussion

The Illumina platform has proven to be an effective technology for transcriptomic research on *Lavandula* (lavender) plants such as *L. angustifolia* ‘Lady’, *L*. *latifolia,* and their natural breed *Lavandula* × *intermedia* ‘Grosso’ [8], as well as *L. angustifolia* ‘JX-2′ [9], and *L. angustifolia* ‘Maillette’ [10]. In this study, in which we used this platform to characterize the transcriptome of *L. pubescens*, clustering and assembly of clean reads provided 124,233 unigenes, and on the basis of annotation using multiple databases, we established that *L. pubescens* unigenes showed closest matches with those from *Sesamum indicum,* followed by *Erythranthe guttata*, *Vitis vinifera*, *Coffea canephora*, *Nicotiana sylvestris*, *Beta vulgaris,* and *Salvia miltiorrhiza*.

The COG database provides a standard system for estimating the evolution and function of coding proteins at the genome scale, and our COG annotations revealed that a majority of the identified unigenes are associated with the functional categories “Translation, ribosomal structure and biogenesis, carbohydrate transport and metabolism,” “post-translational modification, protein turnover, chaperones,” “general functional prediction only,” and “signal transduction mechanisms.” Furthermore, using GO annotation to comprehensively delineate the functions of *L*. *pubescens* unigenes and their products, we found that the predicted unigenes were grouped into the three major GO categories, namely cellular components, molecular functions, and biological processes, with a majority being associated with cells and cell parts, binding and catalytic activity, and cellular and metabolic processes.

With respect to terpene biosynthesis, we identified a total of 14 terpene biosynthetic genes in *L*. *pubescens* (*LpDXS*, *LpMCT*, *LpMCS*, and *LpHDR* in the MEP pathway, *LpAACT*, *LpHMGS*, *LpHMGR*, *LpMVK*, *LpPMK*, and *LpMVD* in the MVA pathway, and *LpIDI*, *LpGPPS*, and *LpSQS*, and *LpGGPP* in the terpenoid biosynthesis pathway), which were confirmed based on NCBI homology BLAST searches. Furthermore, we established that the expression levels of most of the genes associated with terpenoid biosynthesis were higher in the stems and leaves than in the roots, which was consistent with HPLC results, indicating a higher accumulation of ursolic and oleanolic acids in the stems. The level of *LpSQS* was particularly higher in stems and leaves, consistent with the high levels of ursolic and oleanolic acids in the stems and leaves, compared with those in roots. These findings are also comparable to those obtained in previous studies, in which it has been demonstrated that overexpression of SQS enhances the production of phytosterols (stigmasterol and beta-sitosterol) and triterpense (ciwujianosides B, C1, C2, C3, C4, D1, and D2) in *Eleutherococcus senticosus* [32] and phytosterols (stigmasterol, beta-sitosterol, and campesterol) and triterpense (ginsenoside Rb_1_, Rb_2_, Rc, Rd, Re, Rf, and Rg_1_) in *Panax ginseng* [33]. Furthermore, Kim et al. [34] have reported the enhanced accumulation of phytosterol and saikosaponin contents in *Bupleurum falcatum* following the overexpression of SQS [34], whereas Zhou et al. [35] have reported the increased production of ganoderic acids in a submerged culture of *Ganoderma lucidum* subsequent to the overexpression of SQS [35], and similarly in response SQS overexpression, Mitjalili et al. have observed the enhanced production of phytosterols in a hairy root culture of *Withania coagulans* [36].

Carbohydrates are necessary to provide energy for the plant growth and maintenance and precursors or intermediates for the metabolic process. In particular, previous studies reported that the endogenous sucrose level affects secondary metabolite biosynthesis. For example, the enhancement of endogenous sucrose level increased anthocyanin biosynthesis via the activation of flavonoid biosynthesis genes in *Arabidopsis* [37] as well as the pho3 *Arabidopsis* mutant containing great abundance of glucose, sucrose, fructose, and starch, showed increased anthocyanin production [38]. Furthermore, the endogenous abundance of sucrose had a strong positive correlation with galantamine in bulb [20], leaves [20], stems [20], and flowers [39] of *Lycoris radiata*, anthocyanin in fruits of *Morus alba* [30], carotenoids in fruits and leaves of the diploid and tetraploid *Morus alba* [40], and flavones in hairy roots of *Scutellaria baicalensis* [41]. The results from these previous studies were consistent with our results showing that sucrose was positively correlated with ferulic acid, sinapinic acid, oleanolic acid, and ursolic acid, and it was likely to be positively correlated with total phenolic and total flavonoid contents.

In the present study, we identified and quantified oleanolic and ursolic acids in the roots, stems, and leaves of *L. pubescens*, and on the basis of five assay types assessing different radical scavenging activities, demonstrated that *L. pubescens* leaves exhibited strong antioxidant activity. We assume this activity to be associated with the high levels of triterpenoids and total phenolic and flavonoid contents in the leaves of this plant, which is consistent with the findings of previous studies that have demonstrated that triterpenes (oleanolic acid and ursolic acid) have strong scavenging activities against a range of free radicals [42,43]. In addition, it has also been established that diverse phenolic compounds in plants function as natural antioxidants, with a number being reported to show strong antioxidant activity [44].

Many previous studies have shown that lavender species possess strong antioxidant activity. For example, water and ethanol extracts of Ladastacho (*Lavandula stoechas* L.) have been shown to have antioxidant activities comparable to those of commonly used antioxidants, such as butylated hydroxyanisole, butylated hydroxytoluene (BHT), and α-tocopherol [45], whereas ethyl acetate and methanolic extracts from *Lavandula vera* MM cell cultures have also been found to have strong radical scavenging activity compared with those of rosmarinic, caffeic acids, and BHT [46]. Similarly, Robu et al. have reported that high concentrations of ethanol extracts of *Lavandula* flowers (*L. angustifolia* ssp. *angustifolia*, *L. angustifolia* spp. *angustifolia* cv. Munstead, *L. angustifolia* spp. *angustifolia* cv. Hidicote Blue, *L. angustifolia* spp. *pyrenaica*, and *L. hybrida*) show concentration-dependent antioxidant activity in comparison with the positive control antioxidants gallic acid and quercetin [47], whereas extracts of the flowers, leaves, and stalks of lavandin (*Lavandula × intermedia* Emeric ex Loiseleur) and *L. angustifolia* were found to show significant antioxidant potential compared with that of BHT and rosmarinic acid [48].

## 5. Conclusions

In this study, the transcriptome data obtained provide a rich source of functional information on *L*. *pubescens*, which will contribute to further studies on this species, particularly with respect to terpenoid biosynthesis. Notably, the data compiled based on our transcriptome and metabolite analyses have enabled us to gain a better understanding of triterpenoid (ursolic acid and oleanolic acid) biosynthesis and metabolic difference in this plant, and we have demonstrated that ethyl acetate extracts of different plant parts show strong antioxidant activities. Among the plant organs assessed, leaves were found to contain the high contents of triterpenoids (oleanolic and ursolic acids) and total phenolic compounds, which presumably accounts for the observed strong antioxidant activities of leaves compared with those of ascorbic acid and also indicates that the antioxidant capacities of leaves of *L. pubescens* may be attributable to the synergistic interaction of these triterpenoid and phenolic compounds. We believe that the findings of our transcriptomic and metabolic analyses of *L. pubescens*, particularly with respect to ursolic acid and oleanolic acid biosynthesis and its antioxidant properties, will make an important contribution to further studies on this lavender and may have potential commercial and/or traditional medicinal applications.

## Figures and Tables

**Table 1:** The contents of oleanolic acid and ursolic acid detected in the roots, stems, and leaves of *Lavandula pubescens* (mg/g dry weight).

Organ	Oleanolic Acid	Ursolic Acid
Root	0.107 ± 0.00471 ^c 1^	0.120 ± 0.00100 ^c^
Stem	1.20 ± 0.0464 ^a^	1.75 ± 0.0535 ^a^
Leaf	0.963 ± 0.00943 ^b^	1.62 ± 0.0262 ^b^

^1^ values denoted by different letters (a, b, and c, respectively) are significantly different (*p* < 0.05, ANOVA, Duncan’s multiple test).

**Table 2:** Total phenolic and total flavonoid content in the roots, stems, and leaves of *Lavandula* *pubescens*.

Organ	Total Phenolic Content (mg GAE/g DW)	Total Flavonoid Content (mg RE/g DW)
Root	8.08 ± 0.11 ^b 1^	27.76 ± 0.26 ^b^
Stem	3.83 ± 0.07 ^c^	11.71 ± 0.18 ^c^
Leaf	9.58 ± 0.19 ^a^	29.56 ± 0.51 ^a^

^1^ values denoted by different letters (a, b, and c, respectively) are significantly different (*p* < 0.05, ANOVA, Duncan’s multiple test).

## Data Availability

Data is contained within the article and supplementary material.

## Supplementary Materials

The following are available online at https://www.mdpi.com/article/10.3390/antiox10071027/s1, Figure S1: Length distribution of contigs and unigenes in the *Lavandula pubescens* transcriptome. Figure S2: COG functional classification of the *Lavandula pubescens* unigenes, Figure S3: GO annotation of the *Lavandula pubescens* unigenes, Figure S4: Heatmap representing differences in relative metabolite concentrations of roots, leaves, and stems of *Lavandula pubescens*. Increasing and decreasing the contents of metabolites are shown by red and blue color, respectively, Table S1: Summary of unigenes in the *Lavandula pubescens*, Table S2: Summary of the annotation of *Lavandula pubescens* unigenes, Table S3: Comparison of terpene biosynthetic genes of *Lavandula pubescens* with orthologous genes and proteins showing the highest identity, Table S4: Primers designed based on terpene biosynthetic gene sequences.
