# Supplementary material for: Integrated Analysis of Transcriptome and Metabolome and Evaluation of Antioxidant Activities in Lavandula pubescens
Source: Antioxidants (Basel). 2021 Jun 25;10(7):1027. doi: 10.3390/antiox10071027 (PMC8300654; doi:10.3390/antiox10071027)
Supplement: Supplementary file 1 [file antioxidants-10-01027-s001.zip › antioxidants-1264151-supplementary.pdf]

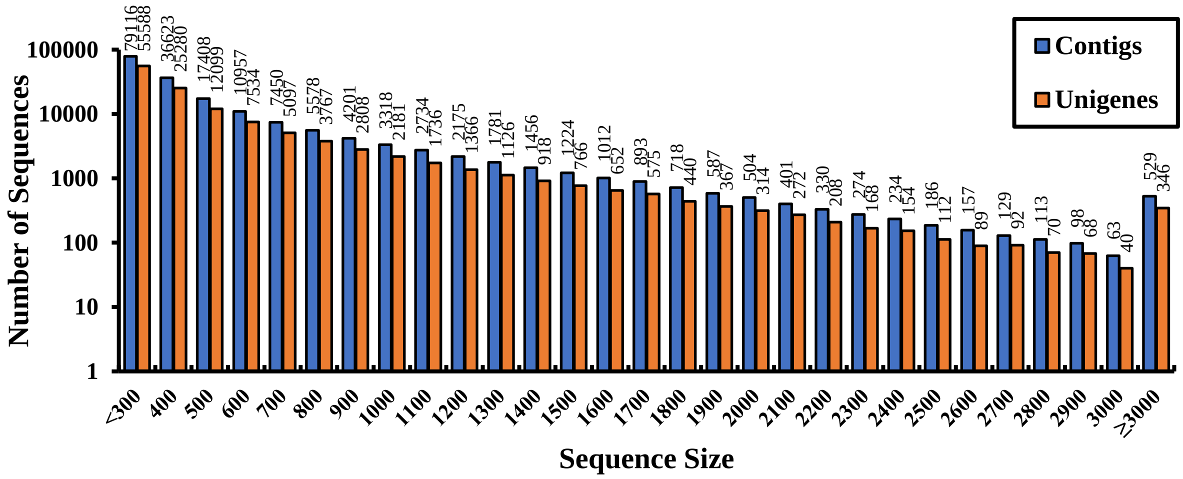

Figure S1. Length distribution of contigs and unigenes in the *Lavandula pubescens* transcriptome.

A	17	RNA processing and modification
B	38	Chromatin Structure and dynamics
C	874	Energy production and conversion
D	192	Cell cycle control, cell division, chromosome partitioning
E	968	Amino acid transport and metabolism
F	371	Nucleotide transport and metabolism
G	1590	Carbohydrate transport and metabolism
H	616	Coenzyme transport and metabolism
I	852	Lipid transport and metabolism
J	1799	Translation, ribosomal structure and biogenesis
K	795	Transcription
L	601	Replication, recombination and repair
M	725	Cell wall/membrane/envelope biogenesis
N	90	Cell motility
O	1464	Post-translational modification, protein turnover, chaperones
P	621	Inorganic ion transport and metabolism
Q	593	Secondary metabolite biosynthesis, transport and catabolism
R	1441	General functional prediction only
S	339	Function unknown
T	1330	Signal transduction mechanisms
U	96	Intracellular trafficing, secretion, and vesicular transport
V	272	Defense mechanisms
W	3	Extracellular structures
X	129	Phage-derived proteins, transposases and other mobilome components
Y	0	Nuclear structure
Z	22	Cytoskeleton

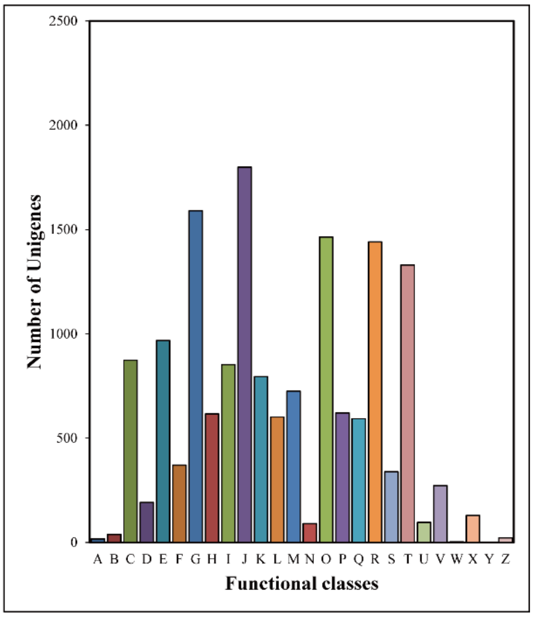

Figure S2. COG functional classification of the *Lavandula pubescens* unigenes.

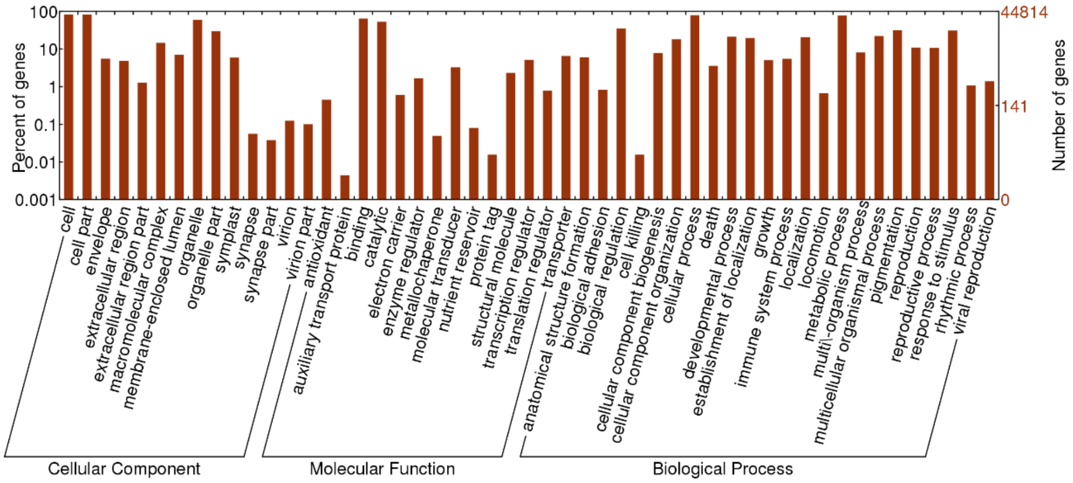

Figure S3. GO annotation of the *Lavandula pubescens* unigenes.

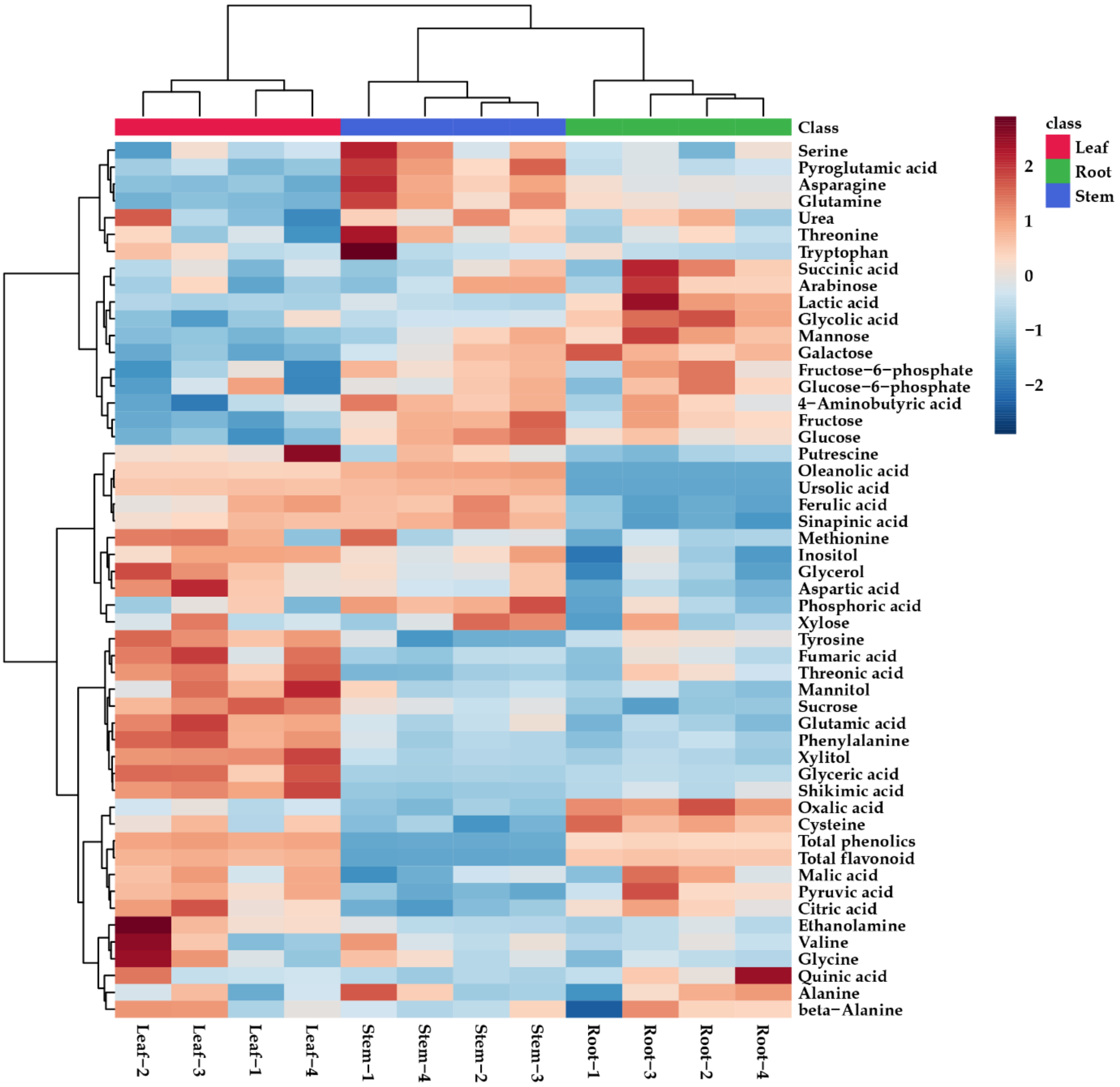

Figure S4. Heatmap representing differences in relative metabolite concentrations of roots, leaves, and stems of *Lavandula pubescens*. Increasing and decreasing the contents of metabolites are shown by red and blue color, respectively.

Table S1. Summary of unigenes in the *Lavandula pubescens*

	Raw reads	Contigs	Unigenes
Total length (bp[1])	9,476,122,928	86,598,482	58,431,429
Number of sequences	124,685,828	180,249	124,233
Average length (bp)	76	480	470
Median length (bp)	76	322	319
Maximum length (bp)	76	11,308	11,308
Minimum length (bp)	76	224	224
N50 (bp)	76	545	522

[1]bp, basepair

Table S2. Summary of the annotation of *Lavandula pubescens* unigenes

	Number of BLASTed unigenes	Ratio (%)
All unigenes	124,233	100
Unigenes BLASTed against Nr	64,993	52.32
Unigenes BLASTed against Nt	20,947	16.86
Unigenes BLASTed against SWISS-PROT	45,750	36.83
Unigenes BLASTed against BRAD	57,951	46.65
Unigenes BLASTed against TAIR	56,604	45.56
Unigenes BLASTed against COG	16,019	12.89
Unigenes BLASTed against GO	44,814	36.07
All annotated unigenes	68,695	55.30

Table S3. Comparison of terpene biosynthetic genes of *Lavandula pubescens* with orthologous genes and proteins showing the highest identity

Genes	Orthologous genes (Accession no.)	Identity (%)	Orthologous proteins (Accession no.)	Identity (%)
LpDXS2	*Lavandula angustifolia* (JX630150.1)	86	*Lavandula angustifolia* (AGQ04154.1)	87
	Isodon rubescens (KT831764.1)	81	*Isodon rubescens* (AMM72794.1)	85
	Prunella vulgaris (MK272806.1)	82	*Plectranthus barbatus* (ALE19960.1)	85
LpMCT	*Isodon rubescens* (MK192294.1)	93	*Isodon rubescens* (QCZ24926.1)	96
	Prunella vulgaris (MK272812.1)	87	*Salvia miltiorrhiza* (AEZ55666.1)	92
	Pogostemon cablin (KF926080.1)	86	*Prunella vulgaris* (QEV81819.1)	91
LpMCS	*Isodon rubescens* (KT948057.1)	92	*Salvia miltiorrhiza* (AFQ95411.1)	83
	Prunella vulgaris (MK272815.1)	89	*Isodon rubescens* (AMW77341.1)	81
	Plectranthus barbatus (KF749265.1)	88	*Phlomis umbrosa* (QEN91944.1)	76
LpHDR	*Lavandula angustifolia* (JX630153.1)	90	*Lavandula angustifolia* (AGQ04157.1)	94
	Isodon rubescens (KT948058.1)	88	*Isodon rubescens* (AMW77342.1)	89
	Salvian miltiorrhiza (JX233817.1)	84	*Salvian miltiorrhiza* (AEZ55670.1)	84
LpIDI	*Isodon rubescens* (KT948056.1)	88	*Salvia miltiorrhiza* (ABV08818.1)	94
	Salvia miltiorrhiza (EF635967.1)	87	*Prunella vulgaris* (QEV81828.1)	91
	Prunella vulgaris (MK272820.1)	87	*Scutellaria barbata* (QEY10174.1)	81
LpAACT	*Isodon rubescens* (KR154220.1)	92	*Isodon rubescens* (ALG00700.1)	93
	Prunella vulgaris (MK272796.1)	89	*Prunella vulgaris* (QEV81803.1)	91
	Scutellaria barbata (MK035028.1)	85	*Sesamum indicum* (XP_011095902.1)	88
LpHMGS	*Lavandula angustifolia* (JX630154.1)	92	*Lavandula angustifolia* (AGQ04158.1)	98
	Isodon rubescens (KX151718.1)	90	*Isodon rubescens* (ANW07497.1)	97
	Salvia miltiorrhiza (FJ785326.1)	87	*Salvia miltiorrhiza* (ACV65039.1)	92
LpHMGR	*Lavandula angustifolia* (JX630155.1)	86	*Lavandula angustifolia* (AGQ04159.1)	93
	Salvian miltiorrhiza (JN831102.1)	82	*Prunella vulgaris* (QEV81808.1)	86
	Osmanthus fragrans (KY451762.1)	79	*Sesamum indicum* (XP_011095902.1)	84
LpMVK	PREDICTED: *Ananas comosus* transcript X1 (XM_020245820.1)	73	*Salvia miltiorrhiza* (AEZ55674.1)	92
	PREDICTED: *Ananas comosus* transcript X2 (XM_020245821.1)	73	*Prunella vulgaris* (QEV81809.1)	91
	PREDICTED: *Ananas comosus* transcript X3 (XM_020245822.1)	73	*Picrorhiza kurrooa* (AGS18028.1)	86
LpPMK	*Scutellaria barbata* (MK035034.1)	85	*Salvia miltiorrhiza* (AEZ55665.1)	86
	Prunella vulgaris (MK272803.1)	85	*Scutellaria barbata* (QEY10159.1)	82
	Osmanthus fragrans (KY451764.1)	77	*Sesamum indicum* (XP_011074642.1)	81
LpMVD	*Isodon rubescens* (MK344321.1)	93	*Isodon rubescens* (QDQ03532.1)	96
	Prunella vulgaris (MK272804.1)	91	*Phlomis umbrosa* (APU50937.1)	94
	Phlomis umbrosa (KU317504.1)	90	*Scutellaria barbata* (QEY10160.1)	94
LpGPPS	*Salvia officinalis* (KY399788.1)	84	*Salvia officinalis* (KY399788.1)	89
	Salvia miltiorrhiza (JX090100.1)	83	*Pogostemon cablin* (AHK06506.1)	89
	Lavandula x *intermedia* (MN641908.1)	82	*Handroanthus impetiginosus* (PIN08988.1)	87
LpSQS	*Prunella vulgaris* (MK272831.1)	89	*Prunella vulgaris* (QEV81838.1)	91
	Osmanthus fragrans (KY992860.1)	84	*Sesamum indicum* (XP_011092839.1)	92
	Camellia oleifera (JX914592.1)	83	*Phtheirospermum japonicum* (GFP85929.1)	88
LpGGPPS	*Plectranthus barbatus* (KP889114.1)	83	*Salvia miltiorrhiza* (ACR19637.1)	86
	Salvia sclarea (MK442922.1)	82	*Plectranthus barbatus* (ALE19959.1)	89
	Lavandula x *intermedia* (MN064858.1)	85	*Salvia sclarea* (QEH04692.1)	86
LpActin	*Lavandula angustifolia* (KY596032.1)	90	*Prunella vulgaris* (AHY94895.1)	99
	Prunella vulgaris (KJ010818.1)	90	*Striga asiatica* (AAC49652.1)	99

Salvia hispanica (*MF621045.1*)	86	*Erythranthe lewisii* (AOR81887.1)	99

Table S4. Primers designed based on terpene biosynthetic gene sequences

Genes	Sequence of forward primer (5'–3')	Sequence of reverse primer (5'–3')	Amplicon length (bp[1])
LpDXS	AGAGAGGGTGGCTCTACTGG	CCACCAACTGACCCTTCCTC	195
LpMCT	AATTTGCATTGCCCGGGAAG	CCTAGTACAGCTGCACCGAC	172
LpMCS	TCGACCTTCATCGACTCGAAC	TCGGTGTCTGGAAAGATCTGC	175
LpHDR	ATCCTAGTTGTTGGCGGATGG	TGTCTCCACCAACTCACCATG	159
LpIDI	CAAACACCTGCTGCAGTCATC	TGTAGAGCATACGCCCCAAAG	165
LpAACT	GGTGTTTGTGCAGAGCTGTG	TGGCTTTCCCTTGCTACCAG	162
LpHMGS	TGACCCGAAGCAGATTGGAC	AATAGTGCTGCAGTCCCACC	165
LpHMGR	CACAGTTGGAGGTGGGACTC	GCCGACATCAGAGAGAGCTC	159
LpMVK	GCAGTGCCATGAATTCCGTC	TGGCTGACCCCCATTGATTG	169
LpPMK	AGCCAATTGGGATCATGAGAGG	ACGTTTCAAGGGCATTTTGAGG	155
LpMVD	TGCTTGAGAGAACTCCGCTC	AAAACGAGGCATGCCAAACC	170
LpGPPS	GGCGTTTCAGTTGATCGACG	TGCCGGATTATCGAAGCCTC	172
LpSQS	CGTGAAAATGAGACGCGGTC	ATGCACCAGAGTCTCTGCAG	188
LpGGPPS	GTCGAGAAGTTGCGGGTTTTC	TTCTCCAATCCGAGCAGCTTC	161
LpActin	TGAGCCACACAGTCCCAATC	TCCCTCACAATTTCCCGCTC	157

[1]bp, basepair
